# 

**Published:** 1876-09

**Authors:** 


### BOOKS AND PAMPHLETS RECEIVED.

### BOOKS.

A Practical Treatise on Materia Medica and Therapeutics. By Roberts Bartholow, M.A., M.D., etc. D. Appleton & Co., 1876.

Medical and Surgical Memoirs, containing investigations on the geographical distribution, causes, natural relations and treatment of various diseases. Vol. I. By Joseph Jones, M.D., etc. New Orleans.

Lectures on Orthopedic Surgery and Diseases of the Joints. By L. A. Sayer, M.D., etc.

A System of Midwifery, etc. Second Edition. By Wm. Leishman, M.D., etc., with additions by Jas. S. Parry, M.D.

Spiritualism and Allied Causes and Conditions of Nervous Derangement. By Wm. A. Hammond, M.D., etc.

Consumption in Australia. By C. E. Reeves, B.A., M.D., etc.

Guy's Hospital Report for 1875.

A Manual of Physiology. By Austin Flint, Jr., M.D., etc.

A Practical Treatise on Diseases of the Eye. By C. B. Carter, F.R.C.S., etc. Edited with test types and additions, by Jno. Green, M.D., etc.

Lectures on Fever. By Wm. Stokes, M.D., etc.

A Contribution to the Medical History of our West African Campaigns. By Surgeon-Major Albert A. Gore, M.D., etc.

The Student's Guide to Dental Anatomy and Surgery. By Henry Sewill, Member of the Royal College of Surgeons, etc.

PAMPHLETS.

Transactions of the Medical and Chirurgical Faculty of Maryland—seventy-eighth Annual Session, April, 1876.

Photo-Micrographs in Histology, Normal and Pathological. By Carl Seiler, M.D., in conjunction with J. G. Hunt, M.D., and J. G. Richardson, M.D. Nos. 1, 2 and 3 of vol. I.

A Surgical Study: Gastrotomy and Gastrostomy. By J. H. Pooley, M.D., etc.

The Cause of Rotation in Lateral Curvature of the Spine. By. A. B. Judson, A.M., M.D., etc.

Thirty-seven Operations of Thoracentesis by Pneumatic Aspiration. By Frank Donaldson, M.D.

A Case of Torpor of the Liver and Bowels from birth, and consequent Persistent Constipation, in a boy six years old. Recovery. By Clarkson Cuthbert, M.D., F. R. C. S. E.

Fifth Annual Announcement of the College of Medicine of Syracuse University, Syracuse, N. Y., 1876–77.

Bellevue Hospital Medical College, city of New York. Annual Circular, 1876–7. Annual Catalogue 1875–6.

Thirty-seventh Annual Announcement of the Woman's Medical College of Pennsylvania, Philadelphia, 1876–7.

Transactions of the Illinois State Dental Society, 1876.

Fifty-sixth Annual Announcement and Catalogue of the Medical College of Ohio, 1876–7.

Thirty-third Annual Report of the Managers of the State Lunatic Asylum, Utica, N. Y., for the year 1875.

A Sketch of the Life and Writings of Louyse Bourgeois, Midwife to Marie De'Medici, the Queen of Henry IV. of France, etc., etc. By Wm. Goodell, A.M., M.D. 1876.

Orthopedic Surgery: Deformities of the Lower Extremities. By Van S. Lindsley, M D., etc.

The Normal Standard of Women for Propagation. By Nathan Allen, M.D., LL.D.

Some Forms of Dyspepsia. Being No. IV. of Vol. II. of "American Clinical Lectures." By Francis Delafield, M.D., etc.

On the Wire Ligature in the Treatment of Ununited Fractures, and in Resections of Bones for Deformity. By William A. Byrd, M.D.

Immobility, or Closure of the Jaw; with Report of Cases. By W. F. Westmoreland, M.D., etc.

1—On Some Practical Points in the Treatment of those forms of Eye Diseases of most frequent occurrence in general practice. 2—A Brief Report of Cases of Sympathetic Ophthalmia and Sympathetic Irritation. 3—Treatment of Acute Iritis in Adults. By A. M. Rosebrugh, M.D.

Eighth Annual Report, etc., of the Toronto Eye and Ear Infirmary, 1875.

Second Annual Report of the Philadelphia Infirmary for Diseases of the Ear. For the year 1875.

